# Using Machine Learning-Based Classification of Postural Stability in Cervicogenic Headache Patients: Predictors and Clinical Implications

**DOI:** 10.3390/life16071061

**Published:** 2026-06-25

**Authors:** Mohamed Abdelaziz Emam, Magda Ramadan, Andras Attila Horvath, Ahmed M. Kadry, Gergo Bolla, Fatma S. Amin, Ahmed S. A. Youssef

**Affiliations:** 1 Basic Sciences Department, Faculty of Physical Therapy, Kafr El Sheikh University, Kafr El Sheikh 33511, Egypt; 2 János Szentágothai Neurosciences Division, Semmelweis University, 1085 Budapest, Hungary; 3 Neurocognitive Research Centre, Nyírő Gyula National Institute of Psychiatry and Addictology, 1135 Budapest, Hungary; andras.horvath.semmelweis@gmail.com (A.A.H.); gergobolla99@gmail.com (G.B.); 4 Basic Sciences Department, Faculty of Physical Therapy, Cairo University, Giza 12613, Egypt; magdaramadan2000@yahoo.com (M.R.); fatma.sedika@gmail.com (F.S.A.); 5 Department of Anatomy, Histology and Embryology, Semmelweis University, 1094 Budapest, Hungary; 6 Faculty of Physical Therapy, Kafr El Sheikh University, Kafr El Sheikh 33511, Egypt; ahmed_tabia@pt.kfs.edu.eg; 7 School of PhD Studies, Semmelweis University, 57 Lehel út, 1135 Budapest, Hungary; 8 Department of Basic Science for Physical Therapy, Faculty of Physical Therapy, Beni-Suef University, Beni Suef 62521, Egypt; 9 Rehabilitation & Diagnostic Sciences Department, Faculty of Medical and Health Sciences, Liwa University, Abu Dhabi P.O. Box 41009, United Arab Emirates

**Keywords:** cervicogenic headache, postural balance, machine learning, pain measurement, sensorimotor system

## Abstract

Background: Cervicogenic headache (CEH) is a secondary headache disorder originating from dysfunction in the cervical spine. In addition to pain, individuals with CEH frequently experience disturbances in postural control and sensorimotor integration, which may compromise functional capacity and quality of life. Conventional clinical assessments typically focus on pain intensity and cervical range of motion; however, these measures often fail to capture the multifactorial mechanisms underlying balance impairments in this population. Machine learning (ML) methods offer the ability to integrate multidimensional clinical data and may provide a more comprehensive approach for identifying patterns of postural stability and the factors influencing balance regulation in CEH. Methods: A secondary analysis was conducted using baseline data pooled from three registered randomized controlled trials, comprising 68 independent participants diagnosed by a neurologist according to the International Classification of Headache Disorders, 3rd edition (ICHD-3). Postural Stability Class served as the primary outcome and was derived from quantitative stability scores categorized as High, Moderate, or Low. Predictor variables included demographic characteristics (age, gender), clinical measures (pain intensity, headache frequency, symptom duration, cervical range of motion), and sensorimotor parameters (center-of-pressure sway and gaze accuracy). Five machine learning algorithms—Random Forest, XGBoost, Support Vector Machine, Logistic Regression, and Gradient Boosting—were trained and evaluated using 10-fold cross-validation with procedures implemented to reduce overfitting. Results: The Gradient Boosting classifier demonstrated the best performance, achieving an accuracy of 0.857 and an F1 score of 0.857, with a cross-validated accuracy of 0.802 ± 0.063. Random Forest and XGBoost achieved accuracies of 0.786. Feature importance analysis identified center-of-pressure sway and pain intensity as the most influential predictors of stability classification, followed by cervical flexion range of motion and gaze accuracy. Demographic variables showed minimal contribution to model performance. Conclusions: Machine learning models were able to distinguish different levels of postural stability in individuals with CEH. The findings highlight the central role of pain and sensorimotor control in balance regulation and suggest that predictive analytics may support precision physiotherapy by enabling rehabilitation strategies tailored to individual sensorimotor profiles.

## 1. Introduction

Cervicogenic headache (CEH) is a form of secondary headache that originates from dysfunction in the cervical spine and typically presents as unilateral head pain. In addition to pain, individuals with CEH frequently experience disturbances in postural stability and sensorimotor function [1]. Increasing evidence suggests that CEH should not be viewed solely as a pain disorder but rather as a condition involving broader alterations in the sensorimotor system. Dysfunction of cervical afferents can disrupt the integration of proprioceptive, vestibular, and visual inputs, which are essential for maintaining head–neck control and stable posture.

The cervical region plays a central role in spatial orientation. A high density of muscle spindles and joint mechanoreceptors continuously provides the central nervous system with information about head position relative to the body. This sensory information is integrated with signals from the visual and vestibular systems to stabilize gaze and maintain balance. When cervical structures are affected by pain or mechanical dysfunction—as occurs in CEH—proprioceptive signaling may become distorted. As a result, the central nervous system receives conflicting sensory inputs, potentially leading to impaired postural control, dizziness, and disturbances in visual–spatial orientation [2,3,4].

Postural instability is therefore considered one of the prominent functional manifestations of CEH. It is commonly assessed using posturography, which quantifies the displacement and velocity of the center of pressure (COP) during quiet standing. Increased COP velocity reflects greater corrective activity by the central nervous system to maintain equilibrium and is typically interpreted as a sign of reduced postural stability. Several studies have reported elevated COP sway velocity in individuals with CEH, particularly under challenging conditions such as standing with eyes closed or on unstable surfaces where reliance on cervical proprioception increases [5,6,7]. Clinically, such balance impairments may contribute to functional limitations and potentially increase the risk of falls, especially in patients with persistent or long-standing symptoms.

Despite the recognized relationship between cervical dysfunction and balance disturbances, traditional clinical assessments often fail to capture the full complexity of these impairments. Measures such as pain intensity, disability scores, and cervical range of motion provide useful clinical information but frequently show weak associations with objective indicators of postural stability. This suggests that balance disturbances in CEH are influenced not only by pain but also by sensorimotor deficits, including impaired cervical proprioception and altered visual–cervical integration [8].

Recent research has therefore focused on specific aspects of sensorimotor dysfunction in CEH. Cervical Joint Position Error (JPE), for example, assesses the accuracy with which individuals can reposition their head to a neutral position following movement and serves as an indicator of cervical proprioceptive acuity. Another area of interest is visual–cervical interaction, commonly evaluated through gaze direction recognition tasks that assess the coordination between eye and neck movements. Deficits in these domains have been associated with both impaired balance and the persistence of symptoms in patients with cervicogenic headache [9]. Taken together, these findings highlight the need for analytical approaches capable of capturing the complex interplay among nociceptive, biomechanical, and sensorimotor factors.

However, identifying the relative contribution of these factors remains challenging using conventional statistical approaches. Balance control in CEH likely arises from interactions among multiple variables—including pain intensity, proprioceptive deficits, and musculoskeletal alignment—making it difficult to determine which factors play the most influential roles in postural stability [10,11]. This complexity may also limit the ability of clinicians to design targeted rehabilitation strategies.

Advances in machine learning (ML) offer promising tools for addressing this challenge. Unlike traditional statistical methods, ML algorithms are well suited for analyzing multidimensional datasets and detecting complex patterns within them. In musculoskeletal research, ML has already been applied to classify movement patterns in individuals with neck pain [12] and to predict treatment outcomes in cervical radiculopathy [13]. Nevertheless, the potential of ML to characterize postural stability patterns in patients with cervicogenic headache has not yet been fully explored.

Therefore, the purpose of the present study was to develop and evaluate supervised machine learning models for classifying postural stability in individuals with CEH using integrated demographic, clinical, and sensorimotor data. By identifying the most influential predictors of stability, this approach may provide insights that support precision physiotherapy and help clinicians tailor rehabilitation strategies according to individual sensorimotor profiles [14].

## 2. Materials and Methods

### 2.1. Participants

Patients (*n* = 68) comprising baseline assessments from three randomized controlled trials were included in this secondary analysis. Cervicogenic headache (CEH) was confirmed by a neurologist (S.R.) in accordance with the International Classification of Headache Disorders, 3rd edition (ICHD-3), uniformly across all three source trials. Additional inclusion criteria were: age between 35 and 49 years; unilateral pain originating in the neck and radiating to the frontotemporal region; pain aggravated by neck movements; restricted cervical range of motion; joint tenderness in at least one upper cervical spine joint (C1–C3); and a headache frequency of at least once per month continuously during the preceding year.

Participants were excluded if they had a history of head or neck injury or surgery, or if they had other musculoskeletal disorders, neurological diseases, metabolic syndromes, hypertension or hypotension, vestibular disorders, or inner ear inflammation.

**Figure 1:**
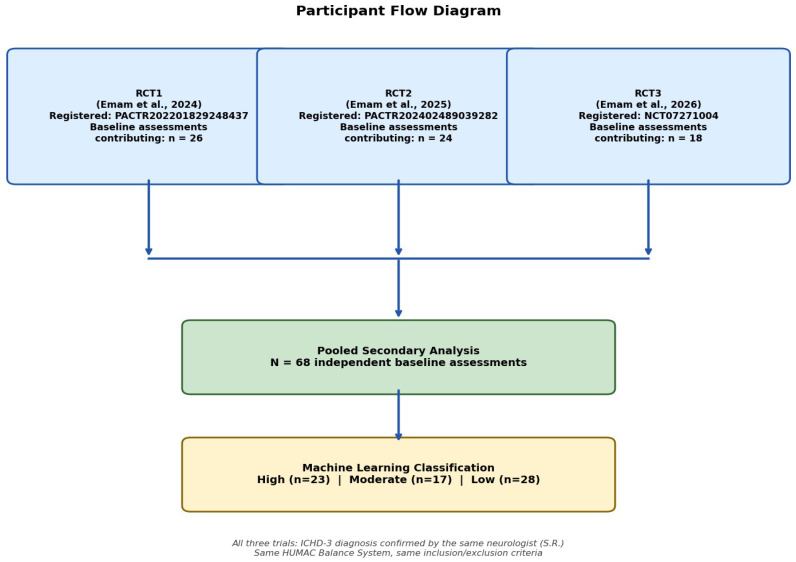
Participant flow diagram showing baseline data extraction from the three source RCTs contributing to the pooled secondary analysis (*n* = 68) [7,9,15].

The dataset used in the present analysis was derived from baseline assessments pooled from three registered randomized controlled trials (RCTs) [7,9,15]. The registration numbers for these trials were: PACTR202201829248437 (RCT1); PACTR202402489039282 (RCT2); and NCT07271004 (RCT3). These three studies recruited participants with similar inclusion/exclusion criteria and collected baseline data using standardized measurement protocols, enabling combined secondary analysis. Baseline assessments contributed by each trial were RCT1 (*n* = 26), RCT2 (*n* = 24), and RCT3 (*n* = 18), resulting in 68 independent participants for this analysis (Figure 1).

### 2.2. Outcome Measures

The primary outcome was the Postural Stability Class, derived from the Postural Stability Score—a standardized metric of the HUMAC Balance System calculated as 100 × (1 − COP excursion area/stability zone area), yielding values from 0 to 100 where higher scores indicate better stability—categorized as High (score ≥ 85), Moderate (score 70–84), or Low (score < 70). Measurements were obtained using the HUMAC Balance System (CSMi, Stoughton, MA, USA) with participants standing barefoot on a compliant foam surface with their eyes closed, a condition that increases proprioceptive demands. Participants stood with their feet shoulder-width apart and their arms resting at their sides for 30 s. Each measurement was repeated three times, and the mean COP velocity (mm/s) was used for analysis. Higher COP velocity values indicate lower postural stability.

Predictor variables included demographic factors (age, gender), clinical variables (headache intensity assessed using the Visual Analog Scale, headache frequency, and symptom duration (hours per attack)), cervical motion parameters (range of motion for flexion and extension), and sensorimotor variables (COP sway velocity measures and gaze accuracy). These variables were selected based on their established associations with proprioceptive dysfunction and balance impairments in individuals with CEH. No missing values were present in the dataset; all baseline measurements were complete across the three source trials. Descriptive statistics for all predictor variables, stratified by postural stability class, are presented in Table 1.

### 2.3. Machine Learning Analysis

The processed data were analyzed using the Python programming language (version 3.10) with the scikit-learn and XGBoost libraries. Continuous variables were standardized using z-score normalization, and categorical variables (group and gender) were numerically encoded. The “group” variable encoded treatment arm assignment (experimental vs. control) within each RCT and was retained as a covariate to account for potential between-group variation at baseline; its ultimately minimal contribution to the model (0.62%) confirmed that classification was driven by sensorimotor and clinical variables rather than study-assignment effects. The dataset comprised 68 independent baseline assessments pooled from three separate RCTs, allowing for between-participant analysis without concerns regarding repeated-measures correlation structures.

Five supervised machine learning classification models were implemented to predict the Postural Stability Class (High, Moderate, or Low): Logistic Regression, Support Vector Machine (SVM), Random Forest (RF), Gradient Boosting (GB), and Extreme Gradient Boosting (XGBoost). To mitigate class imbalance, the Synthetic Minority Oversampling Technique (SMOTE) was applied exclusively within each training fold of the cross-validation procedure, preventing synthetic samples from being generated using test-set observations. The dataset was first divided into a training set (80%, *n* = 54) and a held-out test set (20%, *n* = 14) using stratified random sampling to preserve class proportions. Ten-fold cross-validation was then applied to the training set only for model selection and internal performance estimation. The held-out test set was reserved exclusively for final evaluation and was not used during training or cross-validation.

Hyperparameters were set following established regularization principles for ensemble methods applied to small datasets—specifically, shallow tree depth (max_depth 3–4), low learning rate (0.05), subsampling (0.8), and L1/L2 penalty terms where applicable—to limit overfitting. No automated hyperparameter search was performed, as cross-validated parameter selection on *n* = 68 would produce unstable estimates.

**Figure 2:**
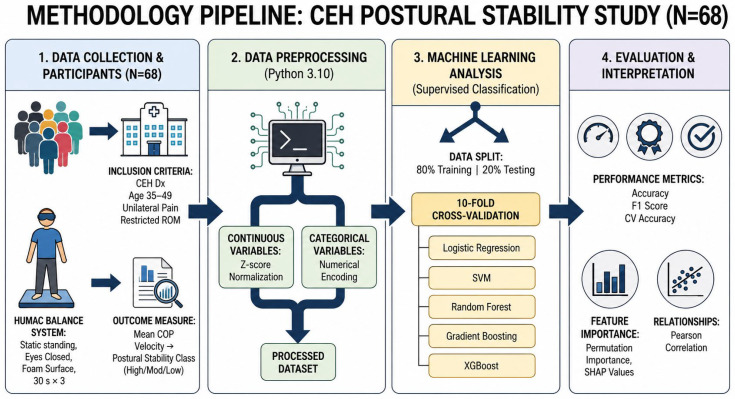
Machine learning workflow. Overview of the data processing and machine learning pipeline used to classify postural stability, including feature preprocessing and model training.

Classification performance was assessed using accuracy, weighted F1 score, and cross-validated accuracy. For the best-performing model, macro-averaged F1 score and balanced accuracy were additionally reported. Feature importance was evaluated using the built-in impurity-based feature importances of the Gradient Boosting classifier, normalized to sum to unity, to facilitate interpretability of predictor contributions. Pearson correlation coefficients were additionally calculated to support interpretation of the relationships between predictor variables and postural stability (Figure 2).

### 2.4. Statistical Analysis

Descriptive statistics were computed for all variables and are reported as mean ± standard deviation (SD). Correlation analyses were conducted to examine associations between postural stability and clinical and sensorimotor variables.

Cross-validation procedures were used to assess the generalizability of the classification models. All statistical analyses were performed using Python libraries including SciPy, scikit-learn, XGBoost, and NumPy. Statistical significance was set at *p* < 0.05.

## 3. Results

### 3.1. Model Training and Evaluation

**Figure 3:**
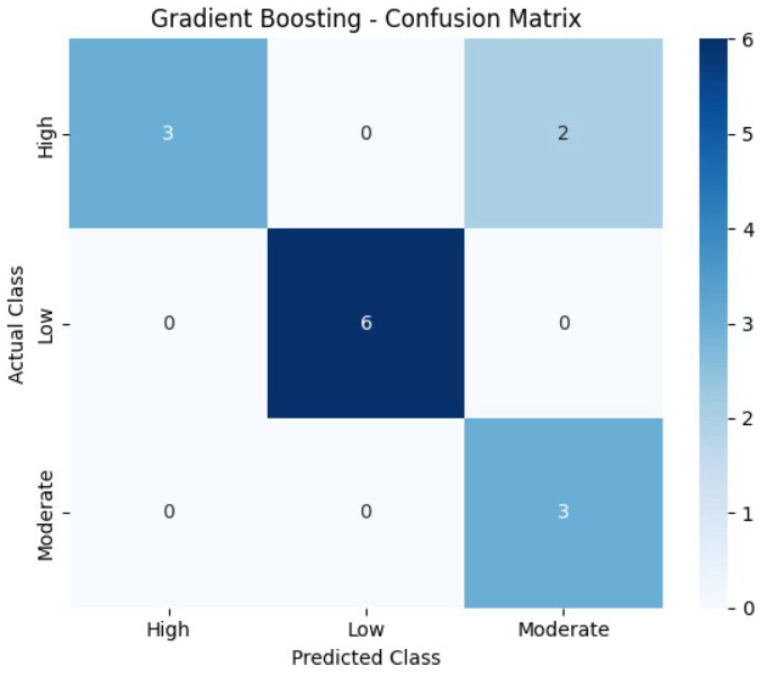
Confusion matrix for the Gradient Boosting classifier on the held-out test set (*n* = 14). The only misclassifications were two High-stability participants predicted as Moderate; the Low and Moderate classes were classified with perfect recall.

A total of 68 independent participants diagnosed with cervicogenic headache (CEH) were included in the analysis, with baseline assessments pooled from three randomized controlled trials. The primary outcome variable, Postural Stability Class, was categorized into three levels based on the Postural Stability Score: High (score ≥ 85), Moderate (score 70–84), and Low (score < 70). The class distribution prior to balancing was: Low (*n* = 28, 41.2%), High (*n* = 23, 33.8%), and Moderate (*n* = 17, 25.0%) (Figure 3).

To mitigate class imbalance, the Synthetic Minority Oversampling Technique (SMOTE) was applied exclusively within each of the ten training folds during cross-validation, ensuring that synthetic samples did not incorporate information from test-fold observations. This approach prevents data leakage and maintains the integrity of performance estimates. Five supervised machine learning classifiers were subsequently trained to predict the Postural Stability Class based on a combination of demographic, clinical, and sensorimotor features. Model performance was assessed using accuracy, F1 score, and cross-validated accuracy, following an 80:20 train–test split with 10-fold cross-validation.

Among the evaluated classifiers, the Gradient Boosting model demonstrated the highest predictive performance, achieving an accuracy of 0.8571, an F1 score of 0.8571, and a mean cross-validated accuracy of 0.8022 ± 0.0633. For the Gradient Boosting model, the macro-averaged F1 score was 0.833 and the balanced accuracy was 0.867, confirming consistent performance across all three stability classes. Random Forest, XGBoost, and Logistic Regression models yielded comparable but slightly lower performance (accuracy = 0.7857, F1 score ≈ 0.78, cross-validated accuracy ≈ 0.70–0.73). In contrast, the Support Vector Machine (SVM) classifier exhibited limited discriminative capability (accuracy = 0.4286, F1 score = 0.4142), indicating that linear decision boundaries were insufficient to capture the underlying class structure of the dataset (Table 2). As a reference baseline, a naive classifier predicting the majority class (Low stability) for all observations would achieve an accuracy of 41.2% (28/68), substantially below the 85.7% achieved by the Gradient Boosting model, confirming that the ML approach captures meaningful patterns beyond class frequency.

### 3.2. Feature Importance Analysis

Feature importance analysis of the Gradient Boosting classifier, corrected for potential overfitting, revealed that the two most influential predictors accounted for more than 67% of the total model importance. Center-of-pressure (COP) sway velocity (34.55%) and pain intensity (32.46%) emerged as the dominant predictors of postural stability class. Secondary contributors included cervical flexion range of motion (16.61%), symptom duration (5.17%), and gaze accuracy (4.72%) (Table 3).

**Figure 4:**
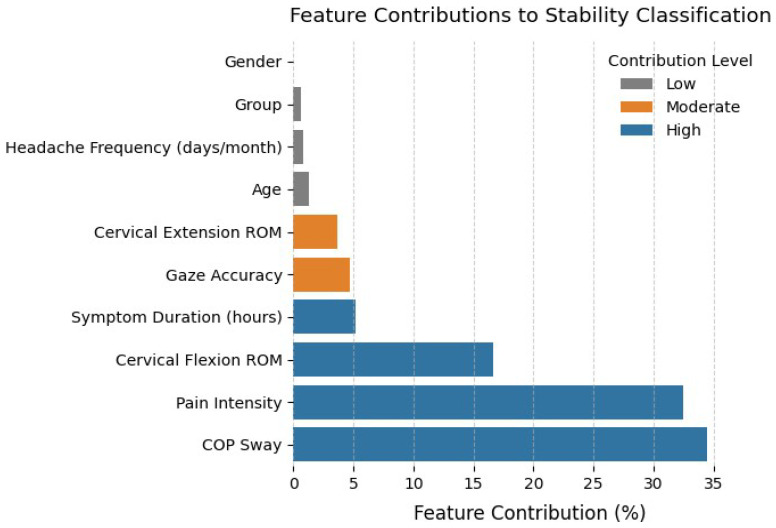
Feature importance from the Gradient Boosting model. Relative contribution of predictors to postural stability classification, showing COP sway velocity and pain intensity as the most influential variables.

Less influential predictors were cervical extension range of motion, age, and headache frequency, whereas categorical variables such as group and gender contributed minimally to model predictions (Figure 4).

These findings emphasize the dominant role of sensorimotor and pain-related mechanisms in differentiating postural stability levels among individuals with CEH. Increased COP sway velocity and pain intensity were associated with reduced stability, whereas improved cervical mobility and gaze control accuracy were linked to enhanced postural performance.

### 3.3. Comparison of Models and Interpretation

Considering predictive accuracy, generalization capability, and interpretability, the Gradient Boosting classifier demonstrated superior performance for categorical postural stability prediction. Its consistent results across training and validation phases indicate effective pattern recognition with minimal overfitting.

The observed hierarchy of feature importance aligns with established biomechanical and neurophysiological models of cervicogenic headache, which emphasize the impact of nociceptive input, proprioceptive disruption, and gaze-related instability on postural control. In contrast, demographic and categorical variables contributed minimally to classification accuracy, supporting the premise that sensorimotor measures provide the most informative predictors of postural dysfunction.

### 3.4. Correlation Analysis

**Figure 5:**
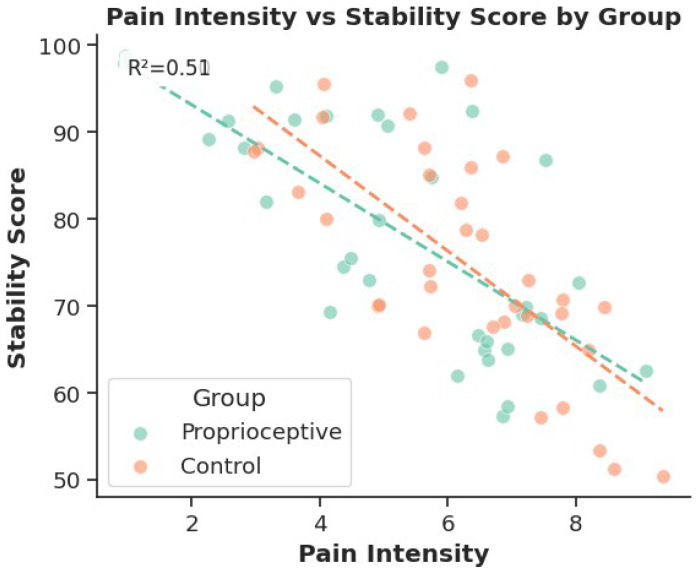
Pain intensity and postural stability. Scatter plot showing a strong negative correlation between pain intensity and postural stability score (r = −0.72). Scatter points represent individual participants, while the colored dashed lines indicate the linear regression trend for each group (green = proprioceptive group; orange = control group). Higher pain intensity was associated with lower stability scores in both groups. The overall coefficient of determination was R^2^ = 0.51.

Correlation analysis further complemented the classification findings. The Postural Stability Score demonstrated strong negative correlations with pain intensity (r = −0.72), indicating that increased pain was associated with poorer postural stability (Figure 5). Moderate positive correlations were observed with cervical flexion range of motion (r = 0.46) and extension range of motion (r = 0.37), suggesting that greater cervical mobility contributed to improved stability. All reported correlations remained statistically significant after Bonferroni correction for multiple comparisons (adjusted α = 0.007; all *p* < 0.001).

**Figure 6:**
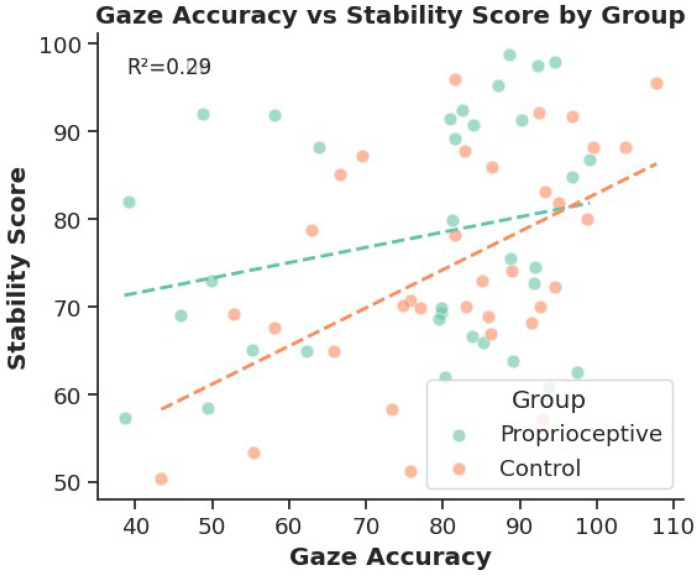
Gaze accuracy and postural stability. Scatter plot showing a positive correlation between gaze accuracy and postural stability score (r = 0.58). Scatter points represent individual participants, while the colored dashed lines indicate the linear regression trend for each group (green = proprioceptive group; orange = control group). Higher gaze accuracy was associated with higher stability scores, with a stronger positive trend observed in the control group. The overall coefficient of determination was R^2^ = 0.29.

Gaze accuracy exhibited the strongest positive association with postural stability (r = 0.58), underscoring the importance of visual–cervical coordination in postural regulation (Figure 6). In contrast, age, symptom duration, and headache frequency showed weak correlations (|r| < 0.20), highlighting their limited contribution to postural stability variance compared with neuromechanical and sensorimotor factors. All reported correlations remained statistically significant after Bonferroni correction for multiple comparisons (adjusted *α* = 0.007; all *p* < 0.001). Correlation analysis was conducted to support interpretation of predictor–outcome relationships and was not used for model training or performance evaluation.

## 4. Discussion

The present study investigated whether machine learning (ML) techniques can be used to classify levels of postural stability in individuals with cervicogenic headache (CEH) using a combination of demographic, clinical, and sensorimotor variables. Overall, the results demonstrate that ML-based models can successfully distinguish between stability profiles in this population, with the Gradient Boosting classifier achieving the highest predictive performance. These findings suggest that data-driven approaches may provide an additional analytical layer to conventional clinical assessment by capturing multidimensional interactions among factors influencing postural control.

An important observation emerging from the analysis is the dominant role of sensorimotor and pain-related variables in the predictive hierarchy. This pattern aligns with contemporary neurophysiological perspectives that describe CEH not solely as a pain disorder but as a condition involving disrupted integration of cervical proprioceptive, vestibular, and visual information [8]. Within this framework, postural instability may represent a functional manifestation of altered sensorimotor processing rather than merely a secondary consequence of pain. The current results therefore support the growing view that balance impairments should be considered a central component of the pathophysiology of cervicogenic headache.

### 4.1. Sensorimotor Contributions to Stability Prediction

The dominance of COP sway velocity in the predictive hierarchy is consistent with the established neurophysiological role of cervical afferent input in postural regulation. COP velocity reflects the frequency and magnitude of corrective adjustments performed by the central nervous system during quiet standing, and increased sway is a recognized marker of reduced postural stability. In the context of CEH, disturbances in cervical afferent input—arising from pain or mechanical dysfunction—can produce sensory mismatch between cervical, vestibular, and visual inputs, leading to inefficient postural strategies and elevated sway during static tasks.

The strong contribution of pain intensity to the model (r = −0.72, *p* < 0.001, Bonferroni-corrected) reflects the well-documented role of nociceptive input in disrupting motor control through reflexive muscle guarding, altered neuromuscular recruitment, and changes in vestibulospinal responses [8]. These adaptations may initially serve protective purposes but can ultimately impair sensorimotor coordination. Taken together, these two dominant predictors suggest that postural stability in CEH is shaped by the interaction between proprioceptive integrity and pain-related modulation of motor control—a relationship that ML approaches are well suited to capture because they evaluate multiple predictors simultaneously rather than in isolation.

### 4.2. Secondary Factors and Sensorimotor Interactions

Although COP sway and pain intensity dominated the predictive hierarchy, additional variables contributed to the classification of stability levels. Cervical flexion range of motion accounted for approximately 16.6% of feature importance and showed a moderate positive correlation with stability (r = 0.46). Reduced cervical mobility may restrict the ability to perform adaptive head movements that assist in maintaining balance, particularly when other sensory inputs become unreliable.

Gaze accuracy also emerged as a relevant contributor (4.7%, r = 0.58), highlighting the role of visual–cervical coordination in postural regulation. Effective gaze stabilization requires precise integration of eye and neck movements. Deficits in this process may impair the ability to maintain spatial orientation, thereby increasing the challenge of postural control.

In contrast, demographic variables had minimal influence on model predictions, contributing less than 3% to the overall importance ranking. While age-related declines in balance have been widely documented in the general population [16], the current results suggest that sensorimotor dysfunction associated with CEH may play a more dominant role than demographic characteristics in determining stability outcomes.

Further insight was provided by the feature importance analysis, which revealed the hierarchical contributions of predictors to postural stability classification. In particular, the combination of high pain levels and elevated COP sway velocity jointly accounted for over 67% of model importance, suggesting that these two variables interact to produce stronger destabilizing effects than would be expected from either in isolation. Conversely, higher gaze accuracy appeared to partially compensate for the negative influence of moderate pain levels. These patterns illustrate the advantage of ensemble ML models in capturing synergistic or compensatory effects that may remain undetected in conventional univariate analyses [17].

### 4.3. Clinical Interpretation of Model-Derived Thresholds

To facilitate translation of these findings into clinical practice, the predictor value ranges corresponding to each stability class are summarized in Table 4. To avoid any ambiguity, the stability classes (High, Moderate, Low) were defined prior to model training using the HUMAC system’s normative score zones (≥85, 70–84, <70). The model was trained to predict these pre-existing classes. The values in Table 4 are the ranges of predictor variables observed within each class in this dataset—they are descriptive summaries, not outputs of the model, and not clinically validated thresholds. These values should be interpreted as indicative reference points only.

The thresholds broadly reflect the relationships observed in the feature importance analysis and emphasize the clinical relevance of sensorimotor and pain-related domains. Previous rehabilitation studies have highlighted the effectiveness of targeted proprioceptive and neuromuscular training interventions for improving balance control in patients with cervical disorders [18]. The present findings support these approaches by providing quantitative indicators that may assist clinicians in identifying individuals who could benefit from such interventions.

### 4.4. Clinical Implications

The findings of this study are preliminary and require external validation before any clinical application can be considered. That said, the classification framework offers a conceptual basis for future precision physiotherapy approaches, in that individuals with low postural stability—characterized by high COP sway velocity, high pain intensity, and reduced cervical ROM—may represent a subgroup that could benefit from rehabilitation targeting proprioceptive retraining, gaze stabilization, and pain modulation. Those with high stability may require different management priorities focused on maintaining sensorimotor performance [19].

A further value of the ML approach, even at this exploratory stage, is its capacity to reveal how predictors interact. The finding that gaze accuracy partially offsets the destabilizing effect of moderate pain levels—identified through the feature importance hierarchy—illustrates the kind of compensatory relationship that is difficult to detect through conventional univariate analysis. Whether these patterns hold in larger, independent cohorts remains to be established.

### 4.5. Limitations and Future Directions

Despite the encouraging findings, several limitations should be acknowledged. First, the analysis relied on secondary data pooled from three randomized controlled trials, each with distinct designs and protocols. While baseline assessments were standardized across the trials, heterogeneity in recruitment populations, measurement techniques, or other methodological factors may have influenced results. Secondary analysis of existing data limits the ability to harmonize variables or control for unmeasured confounders that may impact the relationships observed.

Second, although this analysis comprises 68 independent baseline assessments from three separate trials, the sample size remains modest by contemporary machine learning standards. With an 80:20 split, only 14 participants formed the held-out test set, meaning the single-split point estimates should be interpreted with caution; the 10-fold cross-validated accuracy (0.802 ± 0.063) is the more reliable performance indicator. Larger multi-center datasets would provide more stable parameter estimates, enhance generalizability, and strengthen confidence in the predictive performance metrics, particularly for less frequent stability classifications.

Third, the model was validated internally using cross-validation only. No independent external cohort was available for validation. An attempt was made to obtain additional data from other research groups to enable external validation; however, no data were made available at the time of analysis. The generalizability of the model to other clinical settings or populations therefore cannot be confirmed and should be the focus of future work.

Fourth, applying SMOTE within small training folds may generate synthetic samples with limited diversity, as the synthetic observations will closely resemble the real ones when the training set is small. This approach addresses class imbalance but does not compensate for the fundamental limitation of a small dataset.

Fifth, both the Postural Stability Score (used to derive the outcome classes) and COP sway velocity (used as a predictor) are derived from the same HUMAC balance test session. Although they measure distinct aspects of postural performance—area-based excursion versus velocity of movement, respectively—they are correlated (r = −0.60). This means that COP sway velocity as a predictor carries some inherent information about the outcome class, which may contribute to its high feature importance and should be considered when interpreting the model.

Sixth, the analysis relied on secondary data, which limited the range of available predictors. Clinically relevant variables such as cervical joint position error, anxiety, depression, and current medication use—all known to influence pain perception and postural control—were not recorded in the source RCTs and could not be included. Their omission may have influenced the feature importance hierarchy, and future prospective studies should incorporate these measures. Behavioral adaptations to chronic pain were also not captured [20].

Seventh, the cross-sectional nature of the data precludes conclusions regarding causal relationships between predictors and stability classifications. Longitudinal studies examining changes in these variables throughout rehabilitation would provide valuable insight into the dynamics of sensorimotor recovery.

Future research should therefore focus on validating the proposed model using larger, multi-center datasets and standardized posturography protocols. Integration of wearable sensor technologies may further expand the applicability of stability monitoring in clinical and home-based settings [21]. Such systems could eventually support dynamic predictive models capable of updating stability classifications as patients respond to treatment.

In addition, further investigation into the biomechanical and neurophysiological mechanisms underlying CEH-related instability may help explain the hierarchical importance of predictors observed in the present study. Neuroimaging approaches, for example, could clarify how pain-related cortical plasticity interacts with sensorimotor integration networks involved in postural regulation [22].

## 5. Conclusions

The present study demonstrates that machine learning techniques can be used to classify postural stability levels in individuals with cervicogenic headache based on combined clinical and sensorimotor variables. Among the predictors examined, pain intensity, center-of-pressure sway velocity, and cervical range of motion emerged as the most influential factors associated with stability classification.

By integrating multidimensional clinical data, machine learning models may provide a more comprehensive perspective on the mechanisms contributing to balance impairments in CEH than conventional univariate analyses. These findings are preliminary and require external validation in larger, independent cohorts before any clinical translation can be considered. If validated, such approaches could complement traditional clinical assessment and inform the development of individualized rehabilitation strategies.

## Figures and Tables

**Table 1:** Baseline characteristics of participants stratified by postural stability class (mean ± SD).

Variable	High (*n* = 23)	Moderate (*n* = 17)	Low (*n* = 28)	Overall (*n* = 68)
Age (years)	40.09 ± 4.17	40.18 ± 4.81	40.11 ± 3.92	40.12 ± 4.17
Body mass (kg)	69.94 ± 4.46	70.13 ± 3.51	70.35 ± 5.22	70.16 ± 4.52
Height (m)	1.70 ± 0.05	1.72 ± 0.07	1.70 ± 0.05	1.71 ± 0.05
BMI (kg/m^2^)	24.36 ± 0.76	24.44 ± 0.82	24.38 ± 0.75	24.39 ± 0.76
Pain intensity (VAS, 0–10)	4.38 ± 1.84	5.51 ± 1.40	7.18 ± 1.16	5.82 ± 1.91
Headache frequency (days/month)	11.61 ± 4.04	11.00 ± 2.69	11.86 ± 3.36	11.56 ± 3.43
Symptom duration (hours/attack)	16.75 ± 13.89	16.25 ± 9.94	12.61 ± 11.20	14.92 ± 11.89
ROM Flexion (°)	49.84 ± 7.66	47.33 ± 7.61	43.00 ± 6.93	46.39 ± 7.84
ROM Extension (°)	62.33 ± 8.32	57.80 ± 8.31	55.40 ± 11.14	58.34 ± 9.92
COP sway velocity (mm/s)	19.76 ± 3.86	24.04 ± 5.74	27.37 ± 4.34	23.96 ± 5.59
Gaze accuracy (%)	84.31 ± 14.73	81.80 ± 16.92	73.35 ± 17.32	79.17 ± 16.90

**Table 2:** Performance metrics of the machine learning classification models.

Model	Accuracy	F1 Score	CV Accuracy (Mean ± SD)
Gradient Boosting	0.8571	0.8571	0.8022 ± 0.0633
Random Forest	0.7857	0.7891	0.7264 ± 0.1439
XGBoost	0.7857	0.7891	0.6967 ± 0.1462
Logistic Regression	0.7857	0.7670	0.6945 ± 0.1774
Support Vector Machine	0.4286	0.4142	0.6802 ± 0.1590

**Table 3:** Feature importance derived from the Gradient Boosting classifier.

Feature	Weight	Weight (%)
COP Sway Velocity (mm/s)	0.3455	34.55
Pain Intensity	0.3246	32.46
ROM Flexion	0.1661	16.61
Symptom Duration (hours)	0.0517	5.17
Gaze Accuracy	0.0472	4.72
ROM Extension	0.0369	3.69
Age	0.0133	1.33
Headache Frequency (days/month)	0.0084	0.84
Group	0.0062	0.62
Gender	0.0001	0.01

**Table 4:** Approximate predictor value ranges characterizing each postural stability class in the present dataset. These values are descriptive summaries and should not be interpreted as clinically validated diagnostic thresholds.

Stability	COP Velocity (mm/s)	VAS Pain (/10)	Flexion ROM (°)
High	<2.0	<4	>45
Moderate	2.1–5.1	4–6	35–45
Low	>5.1	>6	<35

## Data Availability

All data generated or analyzed during this study are available from the corresponding author upon reasonable request.
